# Biological nitrate removal processes from drinking water supply-a review

**DOI:** 10.1186/2052-336X-11-35

**Published:** 2013-12-19

**Authors:** Anoushiravan Mohseni-Bandpi, David Jack Elliott, Mohammad Ali Zazouli

**Affiliations:** 1 Department of Environmental Health Engineering, School of Public Health, Shahid Beheshti University of Medical Sciences, Tehran, Iran; 2 School of Civil Engineering, University of Newcastle, Newcastle upon Tyne, NE1 7RU, UK; 3 Department of Environmental Health Engineering, Health Sciences Research Center and Faculty of Health, Mazandaran University of Medical Sciences, Sari, Iran

**Keywords:** Acetic acid, Autotrophic, Denitrification, Ethanol, Heterotrophic, Nitrate removal

## Abstract

This paper reviews both heterotrophic and autotrophic processes for the removal of nitrate from water supplies. The most commonly used carbon sources in heterotrophic denitrification are methanol, ethanol and acetic acid. Process performance for each feed stock is compared with particular reference nitrate and nitrite residual and to toxicity potential. Autotrophic nitrate removal has the advantages of not requiring an organic carbon source; however the slow growth rate of autotrophic bacteria and low nitrate removal rate have contributed to the fact that relatively few full scale plants are in operation at the present time.

## Introduction

Extensive research has focused on the removal of nitrate from groundwater and surface water due to its adverse health effects i.e. methemoglobinemia and possible formation of nitrosamines [1,2]. Today nitrate is a major water pollutant in many areas in the world such as Saudi Arabia [3], India [4], UK [5], North America [6], Australia [7], Morocco [8], Changshu in China [9] and Toyserkan in western Iran [10]. Nitrate pollution is caused by the intensive use of nitrogen fertilizers, crop irrigation with domestic wastewater and use of manure, therefore, it is concern of diffuse pollution [11].

Biological process has recently been applied in the field of drinking water treatment due to efficient performance and problems associated with other nitrate removing processes. Physical and chemical methods such as ion exchange, reverse osmosis, nanofiltration and electrodialysis, all show poor selectivity for nitrate removal [12,13]. As well as, the utility of these processes has been limited due to their expensive operation and subsequent disposal problem of the generated nitrate waste brine [11].These methods are more commonly used for the removal of inorganic substances other than nitrate [13]. The main disadvantage of the ion exchange process is that, it produces a nitrate, chloride and sulphate rich brine as a by-product which is difficult to dispose. Although new improved nitrate selection ion exchange resins are now available, this type of resin produces a residual waste stream, rich in nitrate [14-16].

Some processes such as membranes, adsorption, ion exchange resin and photocatalytic reduction are used for nitrate removal. However, these have some problems that include the need for waste brine disposal and post-treatment in membrane and ion exchange processes; saturated adsorbents in adsorption; low efficiency and high operation cost in photocatalytic processes [17-21]. In some cases biological denitrification may need to be coupled with an ion exchange resin process in order to optimize the overall efficiency of the nitrate removal [22]. In this process nitrate is removed by an ion exchange process. Regeneration of the rich nitrate load resin is carried out in a closed circuit by biological denitrification. A membrane bioreactor (MBR) was investigated for denitrification of nitrate (NO_3_^-^) contaminated drinking water [23].

Biological denitrification has the advantage of harmless nitrogen gas being the major end product, and its use for nitrate removal has been promoted by the European Strategy as reference opposed to the physiochemical treatment alternatives [24-27].

The biological processes for denitrification can be of the fixed-film (attached growth) or suspended growth type. With fixed-film denitrification, the organisms are attached to an inert support media and although various media can be used, the goal is to maximize the surface area available for the biofilm to develop. These include fluidized bed reactors, packed bed reactors, and biofilters comprised of sand, anthracite, activated carbon, calcium carbonate, or sulfur [28].

The biological nitrate removal from drinking water supply first became operation in 1981, at the Chateau-Landon in France [29]. Nowadays there are many full scale heterotrophic and autotrophic biological nitrate removal processes around the world [30-37]. An organic carbon source such as methanol, ethanol or acetic acid is required for cell growth and as the energy source for the heterotrophic bacteria. Most drinking water supplies lack sufficient quantities of organic carbon, required by the bacteria, to efficiently carry out the process of denitrification. Several inorganic compounds, such as sulphur and hydrogen, can also act as electron donors for autotrophic bacteria in place of an organic electron donor.

The type of electron donor is the main difference between heterotrophic and autotrophic biological nitrate removal processes. Removal of nitrate from water supplies, using methanol, ethanol and acetic acid is not well documented. This literature survey reviews the various aspects of denitrification of drinking water, using alternative types of bacteria and different reductants.

### Heterotrophic denitrification

Heterotrophic bacteria can utilize different carbon compounds as electron donors. Commonly available carbon sources are sugar, glucose, acetone, acetic acid, ethanol and methanol [38-41]. A number of researchers developed natural materials (wheat straw, plant prunings etc.) as organic carbon sources for use in heterotrophic denitrification. The method was cost-effective but the pretreatment process was complicated and lengthy [25]. The denitrification rate is strongly affected by the type of carbon source [38-41]. Hamlin et al. showed that denitrification rate as g/day nitrate–N was 670, 670, 680 and 670 for methanol, acetic acid, Starch (Glucose) and Molasses (Sucrose), respectively [38]. Also Xu et al. found that polycaprolactone and polylactic acid were suitable carbon sources for denitrification [40]. In generally, the rate is found to be very low, using sucrose and cellulose 0.07 and 0.008 kg/m^3^.d respectively [42,43]. High rates can be obtained with acetic acid [44,45]. In practice carbon sources for nitrate removal from drinking water, are limited to simple and readily degradable substrates such as methanol, ethanol and acetic acid.

Methanol has been used as a carbon source for denitrification of drinking water reported by Rogalla et al. [32], Ayyasamy et al. [46] and Hall&Zabel [47], whereas, extensive use of ethanol and acetic acid has been reported by Ghararah [48], Mohseni-Bandpi et al. [49], Magram [50], Green et al. [51] and Roennefhart [52]. Ghararah showed that ethanol as a carbon source was given better results as compared with methanol and acetic acid in an anoxic static bed column [48].

An inadequate dose of electron donor may result in a high level of nitrate and nitrite in the effluent whereas, over loading can cause carbonaceous contamination of the effluent which necessitates post-treatment. The effect of the under-dose of methanol has been reported in the UK and involves the production of intermediate nitrite which is more toxic than nitrate. This problem can be solved by re-oxidation of nitrite to nitrate via chlorination of the denitrified water, but this requires the provision of a high chlorine concentration [53].

The heterotrophic denitrification process is applied most extensively because of its high efficiency and the simplicity of the reactors required. However, effluent turbidity increasing due to bacterial growth and excessive organic carbon resulting in secondary pollution make it unfavorable [25].

### Methanol as a carbon source

#### Methanol requirement

Methanol has been the most widely used exogenous carbon source for wastewater denitrification due to the lower cost and the lower bacterial cell yield compared to the other organic carbon sources [38,54-57]. Use of methanol as a carbon source in the process has been termed “safe only at low concentrations” but it is claimed to have potential toxic effects at higher concentrations [58]. The stoichiometric relationships describing this process are written as follows [25,59,60].

Bacteria energy reactions step 1 and 2:

(1) $$6{\text{NO}}_{3}^{-}+2{\text{CH}}_{3}\text{OH}\to 3{\text{NO}}_{2}+2{\text{CO}}_{2}+4H_{2}O$$

(2) $$6{\text{NO}}_{2}^{-}+5{\text{CH}}_{3}\text{OH}\to 3N_{2}+3{\text{CO}}_{2}+3H_{2}O+6{\text{OH}}^{-}$$

Overall respiratory reaction:

(3) $$6{\text{NO}}_{3}^{-}+5{\text{CH}}_{3}\text{OH}\to 3N_{2}+5{\text{CO}}_{2}+7H_{2}O+6{\text{OH}}^{-}$$

In addition to the dissimulation reaction mentioned above, nitrate is also used in cell anabolism forming a compound of the form C_s_H_7_O_2_N. Approximately 40 percent of the methanol and 10 percent of the nitrate are consumed for cell anabolism [61]. However, the actual amount of methanol required in the influent is higher than that obtained from theoretical calculations.

It has been suggested that the optimum M/N ratio required for efficient denitrification obeys the following equation [60].

(4) $$C_{m}=2.47\;\left(N0_{3}-N\right)+1.53\;\left(N0_{2}-N\right)+0.87\;\left(\text{DO}\right)$$

Where: C_m_ = mg/l methanol required for denitrification,

NO_3_-N = Initial nitrate-nitrogen concentration mg/l,

N0_2_-N = Initial nitrite-nitrogen concentration mg/l,

DO = Initial dissolved oxygen concentration mg/l.

Jeris et al. claimed that the actual amount of Methanol required for an efficient denitrification, is 20–25 percent higher than that suggested by other researchers [59,60,62].

(5) $$\begin{array}{l} {\text{NO}}_{3}^{‒}+1.08{\text{CH}}_{3}\text{OH}+H\to 0.065C_{5}H_{7}O_{2}N+0.46N_{2}\\ \;+0.76{\text{CO}}_{2}+2.44H_{2}O \end{array}$$

#### Health effect

The main disadvantage of using Methanol, is the potential toxicity of the residual Methanol in the denitrified effluent water [63,64]. Methanol can cause blindness at a concentration of 100 mg per kg of body mass. It can be lethal at a minimum dose of 340 mg/Kg. It has been argued [58] that, formaldehyde is a toxic by product during the oxidation of methanol. Adriaan, concluded that, methanol was a poor substrate for the denitrification process, and suggested that, ethanol and acetic acid were more suitable as carbon sources for the process [58]. The Uk Department of the Environmental specifies a maximum admissible methanol concentration (MAC) of 0.25 mg/l for distributed water [53].

In contrast to the above conclusions Cara et al. argued that the actual quantities of methanol mg/kg) in denitrified water, would be 4 to 5 orders of magnitude less than the toxic concentration [63]; It has been reported that, no direct evidence exists for cytotoxic [65], toxicological and mutagenic [66] activity at low methanol concentration.

Liessens et al. [61] calculated that drinking water denitrified using methanol would contribute approximately 10 percent to the total average direct intake from all sources. This indicates that, the health risks from methanol in water are negligible when the concentration is within the prescribed limit. So far, there has not been a comprehensive study on the probable long term effects on the human body of methanol intake in low concentrations in the daily diet [61].

It may be noted that biological denitrification using methanol as a carbon source results in the complete dehydrogenation of the organic substrate [25]. Thus no intermediate compound will be accumulated during the process. However, the UKDOE recommended continuous on-line monitoring of the methanol concentration in the denitrified water [53].

Elimination of methanol from the effluent of the denitrification process can be readily achieved. The result of the investigations carried out by Mohseni and Elliott indicate that methanol remaining in the effluent of the denitrification process can easily be oxidised by the biological action of aerobic bacteria [67-69].

#### Denitrification with methanol as a carbon source

Chang *et al.* investigated the performance of an anoxic filter with one inch gravel filter media for nitrate removal from water using methanol as a carbon source. They reported more than 90 percent removal of approximately 20 mg/l influent nitrate at a temperature 12°C. It was however, noted that the denitrified water would need some post treatment [70].

In an investigation by Croll, *et al.,* methanol, ethanol and acetic acid were used as carbon sources. In this study methanol was applied over long periods, while ethanol and acetic acid were used for short periods. Up to 45 mg/l methanol was required to remove nitrate from water having 12 mg/l dissolved oxygen and 13 mg/l nitrate-nitrogen. The denitrified water was reaerated over a cascade aerator and was kept for a retention time of 1 hour to remove residual methanol before final consumption. No operational problems were observed during the treatment and nitrite concentration in the effluent was controlled by adjusting the dose of the carbon source. It was concluded that the cost of using the ethanol and acetic acid for · nitratenitrogen removal would be respectively 25 and 50 higher than for methanol [71]. The use of a fluidised bed with methanol as a carbon source showed that the highest denitrification rate was found to be 205 g (N0_2_ + NO_3_-N)/m^3^.hr, with an average nitrate-nitrogen removal of efficiency 97.3 percent [72,73].

The use of an upward flow, fluidized sand bed using a spring fed stream was investigated by the Water Research Centre in conjunction with Anglian Water at Bucklesham. Methanol was used as an organic carbon source and phosphate was added to satisfy nutrient requirements. The plant was found to be efficient for nitrate removal. Intermittent high nitrite concentrations were however, observed during a one year experimental period. This investigation was followed by Hall *et al.* under support from the Water Research Centre for two years [47]. According to Hiscock *et al.*[74] the plant achieved a removal of 14 mg/l nitrate-nitrogen at a temperature of 2°C. Methanol was used as an organic carbon source with a dose of 45 mg/l*.* To avoid excess carbon entering the end product, the plant operated under a carbon limited condition with a residual 3–5 mg/l remaining in the treated water. The carbon limited operation however, produced occasional nitrite concentrations of more than 2 mg/l in the product water. Consequently, excess chlorine was required for nitrite removal [74,75].

Pilot plant studies, carried out at Tucking Mill by Wessex Water using a 10 *m*^*3*^*/h* fluidized bed for the denitrification of river water, containing 88 *mg/1* N0_3_, have been described by the UKDOE [53]. In this study methanol was used as a carbon source, with a dose of 30 mg/l*.* The TOC and suspended solids of the water increased as a result of the denitrification process. Nitrite production also occurred during winter operation. Additional chlorination was required to eliminate the residual organic carbon and nitrite in the product water.

Use of a semi industrial biological fluidized bed system has been investigated by Liessens *et al.*[61] with a capacity of 40 *m*^*3*^*/h* and methanol as a carbon source at De Blankaart in Belgium for removing nitrate from surface water. The plant achieved a nitrate removal of 9 Kg N03 */m*^*3*^*.d* at 3.5°C. Residual methanol was readily removed by the existing downstream drinking water treatment processes. It was concluded that the heterotrophic denitrification in a fluidized bed reactor using methanol as a substrate was appropriate for surface water at low temperature. Long term stable operation was demonstrated as well as the ability to achieve high volumetric loading at short residence times. The authors noted that whenever conventional water treatment is used downstream, no residual carbon source can reach the distribution system and give rise to bacterial after growth [61].

### Ethanol as a carbon source

#### Ethanol requirement

The two main mechanisms for ethanol oxidation are as follows [76]:

a) Bacterial respiration

(6) $$2{\text{NO}}_{3}^{-}+5C_{2}H_{5}\text{OH}\to 6N_{2}+10{\text{CO}}_{2}+9H_{2}O+12\text{OH}-$$

A dose of 0.31 mg of ethanol is required to remove 1 mg of N0_3_ by bacteria respiration.

b) Bacterial synthesis:

(7) $$\begin{array}{l} 97{\text{NO}}_{3}^{-}+5C_{2}H_{5}\text{OH}\to 5C_{7}H_{5}O_{2}N+75{\text{CO}}_{2}\\ \;+84H_{2}O+46N_{2}+97\text{OH}- \end{array}$$

A dose of 0.38 mg of ethanol is required to remove 1 mg of N0_3_.

The theoretical amount of ethanol needed as a substrate i.e. the stoichiometrical amount is 2 g C_2_H_5_OH per g nitrate-nitrogen. In practice, however, approximately 3 g C_2_H_5_OH per g nitrate-nitrogen is required (0.6-0.7 g C_2_H_s_OH per g N0_3_). Approximately 0.4-0.6 Kg SS of excess biomass is produced per Kg N [52].

The following equations may also be used for calculating the substrate requirement [32].

(8) $$C=\left(N-{\text{NO}}_{3}\right)\times 0.475+\left(O_{2}\right)0.55$$

Where, (N-N0_3_) = Nitrate removal rate mg/l,

O_2_ = Dissolved oxygen in raw water mg/l,

C = Ethanol concentration mg/l.

In an investigation by Green *et al.*[51], the optimum ethanol to nitrate-nitrogen ratio was found to be 2.2. other research showed that the ratio for efficient nitrate-nitrogen removal with minimum combined effluent nitrite and ethanol to be 2.3 [51,77].

#### Health effect

To overcome the possible toxicity problem due to use of methanol as a electron donor in the nitrate removal process, ethanol has been recommended as alternate safe organic carbon source. No limit has been set for ethanol in potable water. Several states have set guidelines or standards for *ethanol* in ambient air but USEPA has suggested a permissible ambient goal of 26 mg/l based on health effects [78].

#### Denitrification with ethanol as a carbon source

Ethanol also was applied in an investigation by Croll *et al.* using a fluidized bed reactor in short periods at doses of 33 mg/l for removing approximately 12 mg/l DO and 13 mg/l NO_3_-N. The ethanol requirement was 0.5 mg ethanol/mg DO and 2 mg ethanol/mg NO3-N [71]. Mekonen et al. was investigated the efficiency of a sequencing batch reactor in denitrification of drinking water with relatively high nitrate concentrations with Ethanol as a carbon source. It was found that ethanol at a COD/N of 2 was sufficient to reduce nitrate concentrations to acceptable levels (<10 mg/L as N). They claimed that a sequencing batch reactor has the potential of being used as an alternative configuration for biological denitrification of drinking water [79].

The use of heterotrophic microorganisms has been described by Roennefhart [52] for removing nitrate from water supplies in the “DENIPOR” process in Germany. Methanol and acetic acid were used as carbon sources initially, followed by ethanol. The bacteriological study showed that the bacteria found in the denitrified water were similar to those in other drinking waters, and consisted mainly of *Pseudomonas* with dominant species being *Pseudomonas fluroscence* and *Pseudomonas putida.* The plant achieved a removal efficiency of 90 to 95% at loading rates of 2.5 to 5.3 kg/m^3^/d. N. Based on good results of pilot scale studies, the first full scale treatment plant is being built of the water works of the towns of Langenfeld and Monheim (Germany) [52].

Rogalla [32] used a pilot-scale packed bed reactor with a mineral medium and ethanol as the carbon source to remove nitrate from groundwater. The results obtained from the pilot scale studies were applied to full scale reactors. Full scale studies at flow rates of 80 *m*^*3*^*/hr* and 1400 *m*^*3*^*/hr* showed that the complete removal of nitrogen and organic compounds were achieved. Denitrified water was passing through a two-layer media. The first layer consisted of aerated activated carbon for the removal of excess carbonaceous pollution, and to increase the available dissolved oxygen, while the second layer was made of fine sand to remove suspended solids [32].

Two full scale biological nitrate removal processes called “Nitrazur” and “Biodent” with capacities 35–70 and 80 m^3^/h respectively have been operated in France. Acetic acid was used as a carbon source for a short period, which then was followed by the use of ethanol [36]. The first plant achieved a nitrate removal efficiency of 72% with an ethanol dose of 3.1 g per g of nitrate-N removed. The average consumption of ethanol varied from 0.65 to 0.75 g per g of nitrate removed. Based on good performance of the “Bioden” reactor, a similar large scale facility in Dennemout near Paris with a capacity 400 m^3^/h was investigated for removal of nitrate and ammonia, using fixed heterotrophic bacteria and ethanol as the electron donor. The influent nitrate and ammonia concentrations were 40–65 and 2–3.5 *mg/l* respectively. The corresponding effluent nitrate and ammonia concentration were 15–17 and *0.01-0.02 mg/l* respectively. The denitrified water was applied to an aerated two-layer sand and activated carbon filter, before ozonation to oxidise residual ethanol and micro pollutants. No residual ethanol and nitrite were observed in the final effluent.

Continuous rotating biological contactors were investigated in Germany for nitrate elimination from potable water using ethanol as a substrate. The plant consisted of two RBC units in series. The first unit operated under anoxic conditions where denitrification occurred, while the second reactor operated under aerobic conditions. Ethanol and nutrients were dosed automatically, regulated by the nitrate concentration of the raw water. No change in chemical composition of the water was observed, except a reduction in the nitrate concentration. The major operational advantage of the combination was found to be continuous elimination of nitrate and the removal of excess ethanol. Problems of nitrite production were only experienced during the initial period of the experiments. To ensure the removal of excess biomass and organic matter, the final product from the denitrification reactor was passed through the filter beds and the disinfection stage of the water treatment works. It was concluded that, the system was capable of being integrated into the standard water treatment process sequence, rather than replacing the existing physiochemical treatment processes [33].

A pilot plant tube reactor, with a capacity of 650 *m*^*3*^*/d* was operated using ethanol as a carbon source in Germany [33]. Considering the high performance of the reactor for removal of nitrate, two full scale reactors each with a capacity of 6100 *m*^*3*^*/d* were made operational in 1992. Denitrified water was subjected to post treatment consisting of aeration, multi-layer activated carbon, pH adjustment, and disinfection with chlorine. In November 1992 the water was fed into the distribution system for drinking purposes. The analysis of water quality showed that, the final effluent conformed to EC drinking water standards.

### Acetic acid as a carbon source

Acetic acid is more readily metabolisabled than methanol and glucose. It has demonstrated advantages over methanol; higher denitrification rate, high buffering capacity and absence of toxic effects and therefore may be suitable to replace the role of methanol in the denitrification process [80-82].

#### Acetic acid requirement

The stoichiometry of acetic acid as a carbon source for the denitrification process was found experimentally to conform to the following equations [36,80]:

(9) $$\begin{array}{l} 0.84{\text{CH}}_{3}\mathrm{COOH}+{\text{NO}}_{3}^{-}\to 0.08C_{5}H_{7}O_{2}N\\ \;+{\mathrm{HCO}}_{3-}+0.3{\text{CO}}_{2}+0.92H_{2}O+0.46N_{2} \end{array}$$

From the equation above approximately 4.1 g acetic acid is required to remove of 1 g N03-N. Dahab [80] discovered that the mean acetic acid (as carbon) to nitrogen removal ratio *(C/N* removal ratio) was 1.5 [80].

The average acetic acid consumption in an investigation by Richard et al. [36], varied from 0.9 to 1.6 g per g of nitrate removed [36]. The acetic acid requirement in an investigation by Croll et al., was 1.2 mg/mg DO and 3.5 mg acetic acid/mg NO_3_-N [71]. The acetic acid to nitratenitrogen *(A/N)* ratio in an investigation by Mohseni and Elliott. was found to be in the range of 4.2 to 4.3 [49,68]. Table 1 presented some of the organic carbon to nitrate-nitrogen ratios reported in the literature.

**Table 1:** Carbon to nitrate-nitrogen ratios for different carbon sources

**Carbon source**	**Carbon to nitrate-nitrogen ratios**	**Type of system**	**References**
Methanol	2.9 mgM^a^/mg NO_3_-N	Rotating biological contactor	[69]
Methanol	2 mgM^a^/mg NO_3_-N	Intensified biofilm-electrode reactor (IBER)	[25]
Methanol	2.6 mgM/mg NO_3_-N	Fluidized bed reactor	[71]
Methanol	2.6 mgM/mg NO_3_-N	Rotating biological contactor	[27]
Methanol	3.1 mgM/mg NO_3_-N	Fluidized bed reactor	[72]
Methanol	3.2 mgM/mg NO_3_-N	Fluidized bed reactor	[61]
Ethanol	2.35 mgE^b^/mg NO_3_-N	Rotating biological contactor	[69]
Ethanol	2 mgE/mg NO_3_-N	Fluidized bed reactor	[71]
Ethanol	2.2 mgE/mg NO_3_-N	Packed bed reactor	[32]
Ethanol	3 mgE/mg NO_3_-N	Fixed film bed reactor	[52]
Ethanol	2.8-3.3 mgE/mg NO_3_-N	Fluidized bed reactor	[36]
Acetic acid	4.3 mgA^c^/mg NO_3_-N	Rotating biological contactor	[69]
Acetic acid	3.5 mgA/mg NO_3_-N	Fluidized bed reactor	[71]
Acetic acid	3.9 mgA/mg NO_3_-N	Packed bed reactor	[80]
Acetic acid	4.1 mgA/mg NO_3_-N	Packed bed filter	[83]
Acetic acid	4 mgA/mg NO_3_-N	Packed bed reactor	[36]

^a^Methanol; ^b^Ethanol; ^c^Acetic acid.

#### Denitrification using acetic acid as a carbon source

Acetic acid as a carbon source was investigated in nitrate removal from drinking water using a packed bed reactor. The process achieved nearly 100 percent nitrate removal efficiency with an influent nitrate-nitrogen concentration of 100 mg/l [80]. Acetic acid was applied in an investigation by Croll *et al.* (1985) using a fluidised bed reactor in short periods at doses of 57 mg/l for removing approximately 12 mg/l DO and 13 mg/l NO_3_-N [71].

A fixed film reactor followed by aquifer recharge for heterotrophic denitrification of groundwater, was investigated in Germany by the DVGW research department [83]. The total process consists of two steps; an above ground technical treatment followed by underground naturally occurring treatment. The denitrifying bacteria were attached to a granular medium in the reactor through which the water is passed with a downward flow. Acetic acid was added as a substrate to provide energy for the microorganisms together with a small amount of phosphate. At influent nitrate concentrations of 55 to 100 mg/l, the nitrate removal rate was found to be 2.5 to 3.5 kg/m^3^.d respectively, having residual acetic acid in the effluent of 1 mg/l. The removal rate however decreased significantly when the reactor was operated under limited acetic acid i.e. 0.1 mg/l instead of 1 mg/l. After 18 months of pilot scale operation and good results obtained with the system, a full scale plant is was erected at Neuss to treat 30–55 percent of the total flow of 800 m^3^/h. The denitrified water was then mixed with the mainstream. Nitrate levels were reduced from the initial level of 55–60 mg/l to 25 mg/l (as N0_3_) [82].

### Autotrophic denitrification

Autotrophic denitrification is gaining growing attention because it does not require an organic carbon as an electron donor. Autotrophic denitrifiers utilize inorganic carbon compounds (e.g., CO_2_, H_2_CO_3,_${\text{HCO}}_{3}^{-}$) as their carbon source. The substrates required for autotrophic denitrifying microorganisms are hydrogen gas and sulphide ion. Energy is derived from the oxidation reactions of inorganic elements such as hydrogen or various sulfur compounds (H_2_S, S, S_2_O_3_). In this process hydrogen ions are produced, indicating that alkalinity is consumed by the reaction. Therefore alkaline, like limestone, is usually added in the sulfur based autotrophic denitrification reactors [24]. The autotrophic micro-organisms in the nitrate removal process are very few in species and characterized by slow growth resulting in low solids production and low efficiency.

### Hydrogen gas as a reductant

The use of hydrogen oxidising micro-organisms for biological denitrification have been described by some reseachers [76,84-86]. It was shown that autotrophic microorganisms such as *Parcoccus* could use molecular hydrogen as a substrate and inorganic carbon such as CO_2_ and HCO_3_ for energy requirements. Nitrate can be removed in the absence of oxygen by acting as an electron acceptor and is reduced to harmless nitrogen gas.

#### Hydrogen requirement

The consumption of hydrogen gas for the denitrification reaction may be described as follows [82]:

(10) $$2{\text{NO}}_{3}^{-}+2H_{2}\to 2{\text{NO}}_{2}^{-}+2H_{2}O$$

(11) $$2{\text{NO}}_{2}^{-}+3H_{2}\to N_{2}+2H_{2}O+2{\text{OH}}^{-}$$

Overall

(12) $$2{\text{NO}}_{3}^{-}+5H_{2}\to N_{2}+4H_{2}O+2{\text{OH}}^{-}$$

0.35 mg H_2_ is required to complete the reduction of 1 mg nitrate nitrogen. Also one mole of *OH*^-^ is released per mole of nitrate nitrogen reduced [87]*.*

#### Denitrification using hydrogen

A full scale autotrophic groundwater denitrification plant, comprising four fixed film up flow nitrate removal reactors containing hydrogen as a substrate was operated at Monchengladbach [30]. The plant was successful in completely removing nitrate-nitrogen. The denitrified water was passed through a double-layer filter to remove solids. The influent nitrate was found to reduce from 80 to 25 mg/l N0_3_. The denitrified water was then blended with 100 m^3^/hr of mainstream and pumped to the distribution system for drinking purposes. Gormen *et al.* that a hydrogenotrophic denitrification reactor was designed for the removal of nitrate from aquaria, showed that during batch experiments removal rates up to 36 mg N/l reactor per day whereas, during a 7 day aquarium test, a nitrate removal rate up to 18.5 mg N/l reactor per day [88]. A large scale pilot plant using autotrophic bacteria with hydrogen as a substrate showed that the plant achieved more than 97% nitrate- nitrogen removal efficiency with influent nitrate concentration of 18 mg/l*.* The hydrogen consumption was 0.48 mg per mg of nitrate-nitrogen removed. The denitrified water after aeration was passing through 2-layer filtration and disinfected by UV light to remove solids and micro-organisms. The final effluent was good of bacteriological and chemical quality [89].

### Sulphur and sulphide ions as a reductant

#### Sulphur requirement

In addition to hydrogen gas, sulphur and its derivates can also be used as an electron donor for autotrophic bacteria [31,90-95]. Autotrophic bacteria such as *Thiobacillus denitrificans*[26,96] and *Thiomicrospira denitrificans*[97] have been used sulphur as a substrate for removing nitrate from water supplies according to the following reaction:

(13) $$5S+6{\text{NO}}_{3}^{-}+2H2O\to 3N_{2}+5{\text{SO}}_{4}^{2-}+4H^{+}$$

In addition to inorganic nitrogen gas the by-products; sulphate, hydrogen ions and biomass, are also formed [98]*.* Batchelor and Lawrence have conducted kinetic studies with elemental sulfur. The stoichiometric equation for the reduction of nitrate using elemental sulfur proceeds as follows [90]:

(14) $$\begin{array}{l} 55S+50{\text{NO}}_{3}^{-}+38H_{2}O+20{\text{CO}}_{2}+4{\text{NH}}_{4}^{+}\\ \;\to 4C_{5}H_{7}N_{2}O+25N_{2}+55{\text{SO}}_{4}^{2-}+64H^{+} \end{array}$$

#### Denitrification using sulphur

Sulfur-based autotrophic denitrification has been studied in the treatment of drinking water [93,99]. The use of sulphur/limestone as a substrate has been investigated at pilot scale in the Netherlands for removing nitrate from groundwater [100,101]. The system was designed with an up flow filtration rate of 0.25-0.5 m/h. The effluent nitrate varied from 1–2 mg/l. On the basis of pilot scale results, a full scale facility with a capacity of 35 m^3^/h was constructed. The plant comprised a vacuum deaerator for removal of nitrogen and oxygen, a slow sulphur/limestone filter for the denitrification process, a cascade for aerating the water and an infiltration pond for collecting the denitrified water. It was noted that the combination of vacuum deaeration and sulphur/limestone filter could offer a simple process for nitrate removal from groundwater. Based on good performance a 100 m^3^/hr full scale plant was constructed [91]. Darbi *et al.*[100] conducted a study of nitrate removal by using sulphur and limestone autotrophic denitrification, by Thiobacillus denitrificans. The influent NO_3_-N concentration was 94, 57 and 10 mg/l with a maximum hydraulic retention time of 33 d. It was observed that nitrate removal efficiency was >95% at Sulphur: Limestone ratio of 3:1 [102]. Similar study that the influent NO_3_ -N concentration was 30 mg/l with a hydraulic retention time of 30 d showed that nitrate removal efficiency was 95 to 100% with alkalinity control and 80 to 85% without alkalinity control [103].

The autotrophic denitrification process in a lab scale up flow bio filter by using sulfur-limestone indicated that nitrate removal rate was about 90% at the hydraulic retention time (HRT) of 3 hr and temperature of 20–25°C. it was showed that autotrophic denitrification process with sulfur-limestone as the electron donor was feasible to remove the nitrate and nitrite, especially from the low concentration water such as eutrophicated surface water, underground water, or wastewater treatment plant effluent [24].

The results of the full scale plant conform to the pilot scale plant. Nitrate removal in autotrophic denitrification is accompanied by the production of hydrogen ions thus lowering the pH level. pH adjustment is therefore necessary to maintain the optimum pH range for bacterial activity between 6.4 to 6.8. Consequently, limestone granules are added to sulphur to maintain the pH value during the denitrification process [101]. A summary of full scale and pilot scale heterotrophic and autotrophic nitrate removal processes are shown in Table 2.

**Table 2:** Lists of some pilot and full scale heterotrophic and autotrophic denitrification from drinking water

	**Process**^**a**^	**Electron donor**	**Capacity (m**^**3**^**/hr)**	**Influent NO**^**3**^**-N(mg/l)**	**NR**^**b **^**(Kg/m3)**	**Efficiency (%)**	**References**
Heterotrophic”	RBC(pilot Scale)	Methanol	0.15	40	0.1	93	[69]
	IBER(Pilot Scale)	Methanol	-	50	-	97	[25]
	MBR(Pilot Scale)	Methanol	-	200	-	99	[23]
	FBR(Full Scale)	Methanol	40	60-80	0.18	96	[61]
	RBC(Pilot Scale)	Methanol	-	15-20	-	91-93	[27]
	RBC(pilot Scale)	Ethanol	0.15	40	0.9	91	[69]
	“Nitrazur “(Full Scale)	Ethanol	35	16.3	-	72	[36]
	“Biodenit”(Full Scale)	Ethanol	400	14.7	0.41	74	[32]
	Batch-bio-film carrier (pilot scale)	liquorice (Glycyrrhiza glabra)	-	-	-	87	[104]
	Batch-bio-film carrier (pilot scale)	Giant reed (Arundo donax)	-	-	-	100	[104]
	RBC(pilot Scale)	Acetic Acid	0.15	40	0.11	98	[69]
	FBR(Full Scale)	Ethanol	254	-	4.35	-	[33]
	Fixed Film(Full Scale)	Acetic Acid	800	12-22	2.5-3.5	70	[83]
	In-Situ Treatment	Sucrose	50	13.5	0.07	10	[42]
	In-Situ Treatment	Cellulose	60	13	0.008	20	[43]
Autotrophic	Fixed Bed(Full Scale)	Sulphur	-	18.1	-	94	[31]
	SLAD Process (Pilot Scale)	Sulphur	-	10-94	-	>95	[102]
	SLAD Process (Pilot Scale)	Sulphur	-	30	-	95-100	[103]
	Upflow Biofilter (Lab Scale)	Sulphur	-	10-100	-	95	[24]
	Packed-bed bioreactor	Sulphur	-	18 mmol/L	-	95.9	[99]
	Fixed Bed (Full Scale)	Hydrogen	35	18.1	0.6	95	[30]
	Fixed Bed (Pilot Scale)	Hydrogen	50	18	0.85	97	[89]

^a^Rotating Biological Contactor(RBC); Intensified Biofilm-Electrode Reactor (IBER); Membrane Bioreactor(MBR);Sulphur/limestone autotrophic denitrification (SLAD); Fluidized Bed Reactor(FBR).

^b^Nitrate Removal Rate.

## Conclusions

The following main conclusions may be drawn from this literature survey.

i. The use of biological denitrification for removal of nitrate from drinking water is well established. Nowadays there are many full scale biological nitrate removal process, hetrotrophic as well as autotrophic in European countries.

ii. The main sources of carbon source for heterotrophic denitrification are methanol, ethanol and acetic acid. Methanol was found to be cheapest but with possible toxic effects. The use of ethanol is becoming more popular. However acetic acid was found to be more effective in removal of nitrate with low intermediate nitrite in the effluent.

iii. Heterotrophic denitrified water requires post treatment to eliminate residual carbon source from the drinking water and to remove undesirable organic matter which would sustain the regrowth of micro-organisms in the distribution system.

iv. Autotrophic denitrification does not require organic carbon, some inorganic compounds such as hydrogen gas and sulphur for electron donor and bicarbonate or carbon dioxide in the water as a carbon source.

v. Autotrophic denitrified water also needs post treatment for degasification and for removal of biomass.

vi. Autotrophic growth rate is lower than heterotrophic; therefore, the sludge production is low but the efficiency of nitrate removal also lower.

vii. Autotrophic denitrification control is more complex than hetrotrophic, because of the three phase (gas, liquid and solid) process.

## Competing interests

All authors declare that they have no competing interests.

## Authors’ contributions

MAZ has searched, completed and finalized the draft of the manuscript. AMB supervised the study and performed the first draft. DJE was advisor of the study. All authors read and approved the final manuscript.
